# Recent Advances in Fibrous Materials for Hydroelectricity Generation

**DOI:** 10.1007/s40820-024-01537-8

**Published:** 2024-09-30

**Authors:** Can Ge, Duo Xu, Xiao Feng, Xing Yang, Zheheng Song, Yuhang Song, Jingyu Chen, Yingcun Liu, Chong Gao, Yong Du, Zhe Sun, Weilin Xu, Jian Fang

**Affiliations:** 1 College of Textile and Clothing Engineering, Soochow University, Suzhou, 215123 People’s Republic of China; 2 National Engineering Laboratory for Modern Silk, Soochow University, Suzhou, 215123 People’s Republic of China; 3 State Key Laboratory of New Textile Materials and Advanced Processing Technologies, Wuhan Textile University, Wuhan, 430200 People’s Republic of China; 4 Institute of Functional Nano & Soft Materials (FUNSOM), Soochow University, Suzhou, 215123 People’s Republic of China; 5 Department of Electrical and Computer Engineering, University of Massachusetts, Amherst, MA 01003 USA; 6 Department of Materials, Imperial College London, South Kensington Campus, London, SW7 2AZ UK; 7 College of Textile Science and Engineering, Zhejiang Sci-Tech University, Hangzhou, 310018 People’s Republic of China; 8 School of Materials Science and Engineering, Shanghai Institute of Technology, 100 Haiquan Road, Shanghai, 201418 People’s Republic of China

**Keywords:** Hydroelectricity, Fibrous material, Streaming potential, Ion diffusion

## Abstract

Depleting fossil energy sources and conventional polluting power generation pose a threat to sustainable development. Hydroelectricity generation from ubiquitous and spontaneous phase transitions between liquid and gaseous water has been considered a promising strategy for mitigating the energy crisis. Fibrous materials with unique flexibility, processability, multifunctionality, and practicability have been widely applied for fibrous materials-based hydroelectricity generation (FHG). In this review, the power generation mechanisms, design principles, and electricity enhancement factors of FHG are first introduced. Then, the fabrication strategies and characteristics of varied constructions including 1D fiber, 1D yarn, 2D fabric, 2D membrane, 3D fibrous framework, and 3D fibrous gel are demonstrated. Afterward, the advanced functions of FHG during water harvesting, proton dissociation, ion separation, and charge accumulation processes are analyzed in detail. Moreover, the potential applications including power supply, energy storage, electrical sensor, and information expression are also discussed. Finally, some existing challenges are considered and prospects for future development are sincerely proposed.

## Highlights

- Fundamental principles and characteristics of fibrous materials-based hydroelectricity generation (FHG) are thoroughly reviewed.
- Fabrication strategies and advanced functions of FHG are discussed and summarized in detail.
- Challenges and perspectives of the next-generation development of FHG are discussed.

## Graphical abstract

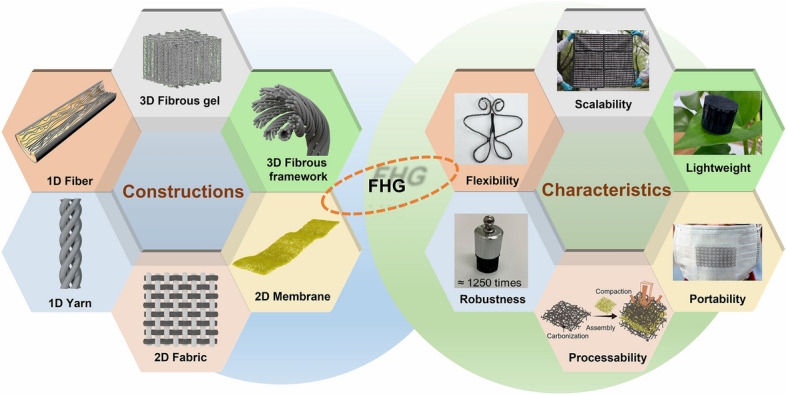

## Introduction

**Fig. 1:**
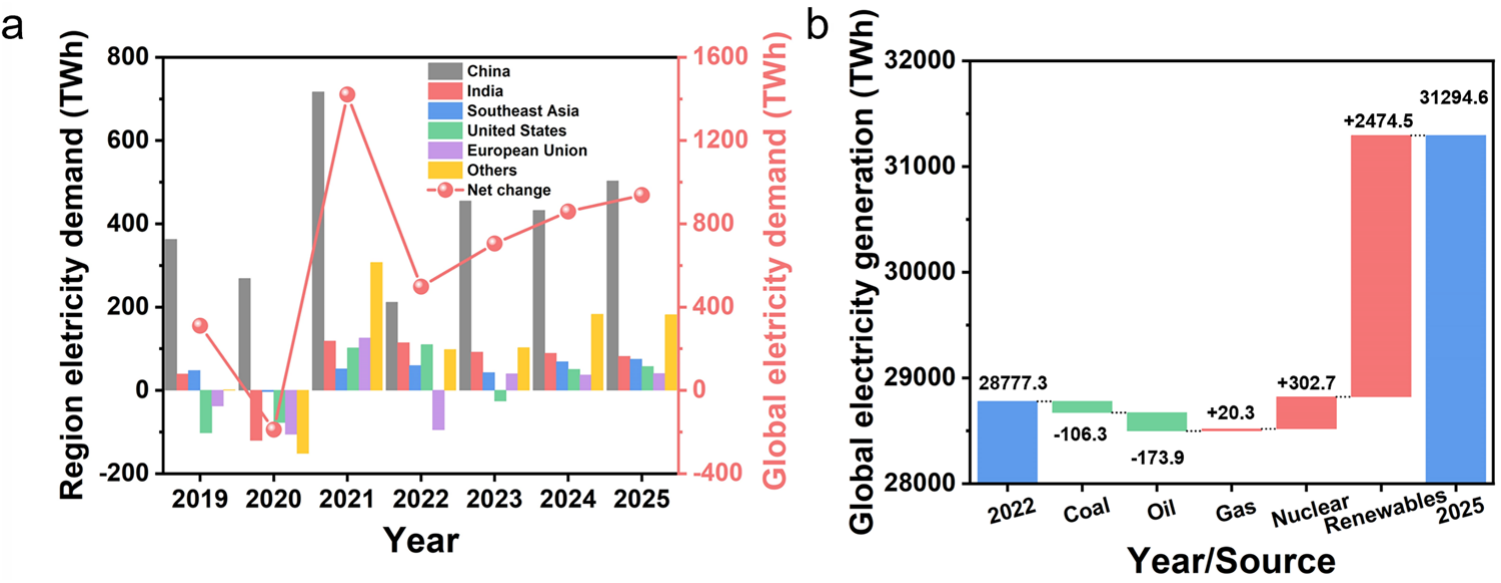
**a** Year-on-year change in electricity demand by region (2019–2025). **b** Changes in global electricity generation by source (2022–2025). The data is obtained from the International Energy Agency

The depletion of fossil energy sources and the environmental pollution resulting from over-exploitation have become a significant threat to the future of mankind [1–3]. There is an urgent need for effective solutions to cope with growing global electricity demand (Fig. 1a). Recently, many countries have expanded their research, development, and utilization of clean energy [4–6]. Low-emission renewables and nuclear energy will dominate (over 90%) the growth of global electricity supply by 2025 (Fig. 1b). Renewable energy generation systems such as triboelectric nanogenerators, photovoltaic generators, and thermoelectric generators have been widely developed through green approaches [7–10]. In contrast to mechanical energy, solar radiation, and heat gradient with characteristics of intermittency and variability, water exists almost everywhere in nature and constitutes a perpetual water cycle in various forms [11–13]. There is a tremendous amount of energy during water cycling. Specifically, the energy that can be converted during the evaporation or condensation of one gram of water is equivalent to the energy contained in one AAA battery (2.6 kJ) [14]. Therefore, hydroelectricity generation (HG) in the water cycle through water–solid interactions has been extensively developed [15–17].

HG with convenient construction, efficient output, and universal applicability can be driven from various forms of water including moisture, evaporation, droplet, wave, and flow [18, 19]. Water molecules with hydrogen and oxygen atoms attract each other via weak hydrogen bonds. The weak bond between the two hydrogen atoms with a tiny dissociation energy of 0.16 eV ensures a dynamic equilibrium between the formation and destruction of hydrogen-bonded water dimers [20, 21]. HG is obtained through the direct interaction between ionic water and chargeable nanostructure through electronic movement. An electric double layer (EDL) consists of an inner immobile Stern layer and an outer diffusion layer that emerges spontaneously owing to coulomb interactions [22]. The Stern layer is composed of an inner Helmholtz plane and an outer Helmholtz plane. The inner Helmholtz plane closest to the electrode is made up of adsorbed ions and solvent molecules, which are impacted by both electrostatic and chemical interactions. The outer Helmholtz plane denotes a charged plane formed by solvated ions, where solvated ions interact with electrode surfaces by long-range electrostatic forces. The diffusion layer consists of abundant free ions in solution and is subject to electrostatic and thermal motions [23, 24]. The EDL between solids and liquids is the main interface for ionic-electronic coupling and plays a key role in controlling electric field effects, ion transport, and surface interactions [25, 26].

In 2009, Galembeck’s group declared that charges of atmospheric water exchanged with the solid surfaces during adsorption and desorption. Later this phenomenon was named hydrovoltaic [27–29]. It has been a decade since Qu’s group introduced the first moisture-induced HG device based on oxidized graphene in 2015 and propelled its development with substantive works [30, 31]. Recently, HG has been considered a promising and revolutionary technology that directly acquires green and renewable energy from global water cycles [32, 33]. Thousands of relevant scientific researches have been conducted in the past decade (data from Web of Science). Fibrous materials with abundant material resources, diverse manufacturing technologies, and flexible constructions demonstrate unique, superior, and extensive characteristics [34, 35]. Specifically, fibrous materials with different dimensions can be divided into 1D fiber, 1D yarn, 2D fabric, 2D membrane, 3D fibrous framework, and 3D fibrous gel. In 2016, a porous carbon film with different functional group contents on two sides was innovatively fabricated by Zhou’s group for potential generation [36]. In 2020, Wang’s group proposed that a large-scale electricity generator based on polytetrafluoroethylene film can instantaneously power 400 commercial light-emitting diodes with several droplets [37]. Recently, numerous efforts have been invested in constructing fibrous materials-based hydroelectricity generation (FHG) systems with unique morphologies, rich functionalities, and excellent performances [38–40]. FHG can be divided into two general types: streaming potential and ion diffusion depending on the source of migrated charges during water–solid interactions [41, 42]. Fibrous materials can be designed to acquire outstanding performance during water harvesting, proton dissociation, ion separation, and charge accumulation processes [43, 44]. Besides, FHG with distinctive cost-effectiveness, scalability, processability, and durability shows outstanding practical prospects [45, 46].

To date, the development of FHG has achieved great progress owing to significant research efforts that have triggered a boom in advanced materials, constructions, techniques, and mechanisms [47–49]. Recent reviews have discussed the development of HG based on different water sources and functionalized loadings. However, a systematic review of the evolution and progress of FHG is necessary but lacking. Hence, we propose to summarize developments of FHG and reveal insights into principles/procedures of hydrovoltaic generation. The prospective analyses and insights are critical to driving FHG practicality. In this review, recent advances in FHG have been analyzed, categorized, summarized, and generalized. First, the design principles and mechanisms of FHG are presented. Then, the fabrication strategies, characteristics, and advanced functions of fibrous materials in FHG are highlighted in detail. Next, the interesting and promising application scenarios of FHG are demonstrated. Finally, the summary and prospects of FHG are meticulously proposed. Overall, this comprehensive review not only introduces the development of FHG but also guides an appropriate direction for future research.

## Mechanisms and Design Principles of FHG

**Fig. 2:**
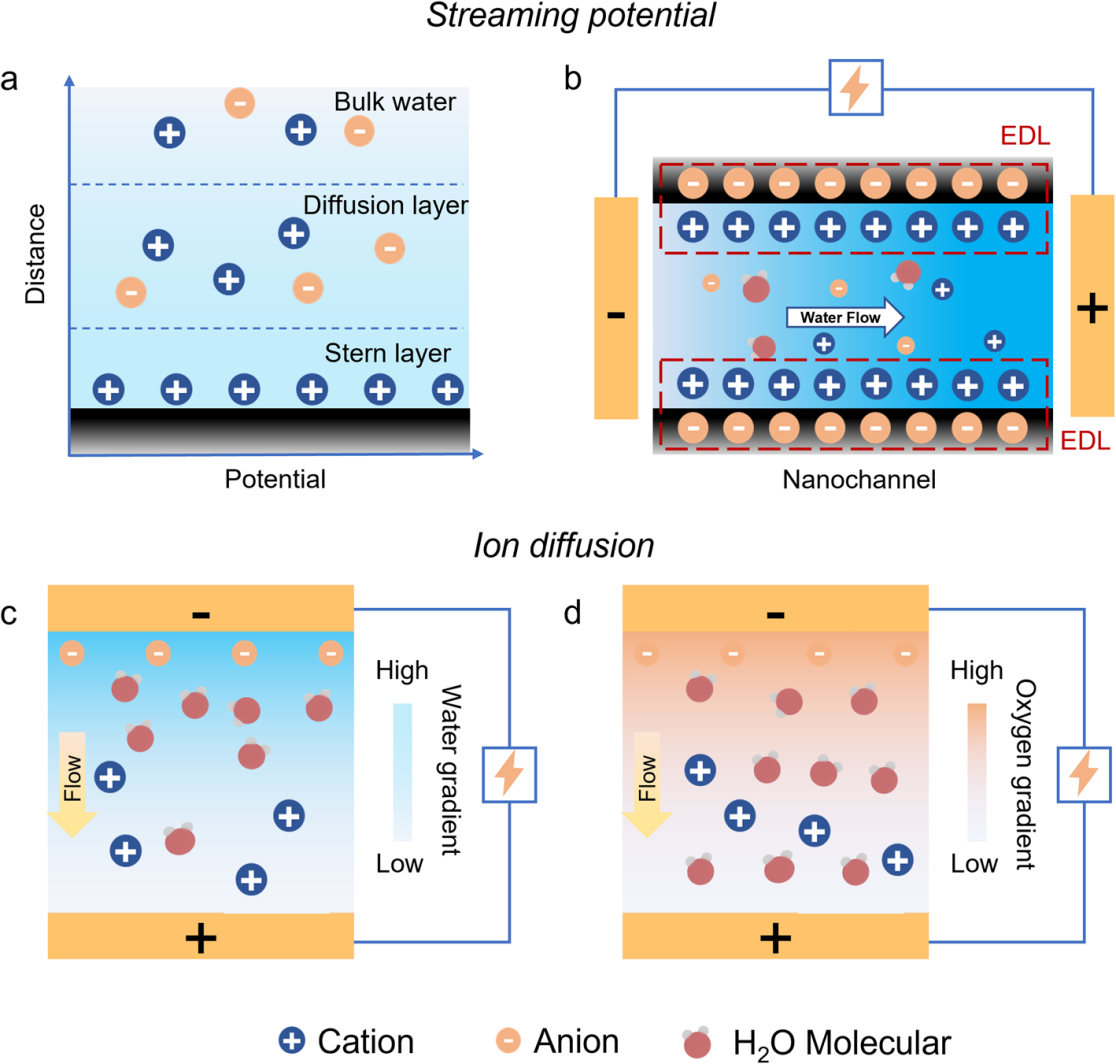
Schematic of streaming potential and ion diffusion-induced FHG. Schematics of **a** EDL at the solid-water interface and **b** the streaming potential within a nanochannel. Schematics of **c** the water gradient mode and **d** the oxygen functional group gradient mode

Numerous FHG devices driven by various forms of water including moisture, evaporation, droplet, wave, and flow have been developed in the last decade [50]. Essentially, the operation mechanisms of FHG can be categorized into streaming potential and ion diffusion according to the source of migrated charges during water–solid interactions (Fig. 2) [51, 52]. Both mechanisms may co-exist in the FHG system and contribute simultaneously to electricity generation. The design principles need to be optimized from both external and internal perspectives to obtain superior performance [53].

### FHG Mechanism

As for steaming potential-induced FHG, an EDL will form at the liquid–solid interface when ionic water contacts the charged solid materials. The EDL consists of an inner immobile Stern layer and an outer diffusion layer (Fig. 2a) [54, 55]. Ions in the Stern layer are tightly attached to the solid surface through electrostatic attraction, and abundant counterions are weakly attracted to the surface charge through coulomb force [56, 57]. The nanochannels with overlapping EDLs repel the co-ions and allow counter-ions to pass through. The unique charge selectivity results in a strong electric field and sharp potential gradient within nanomaterials [58, 59]. The boundary between the Stern layer and diffusion layer is the shear plane, the electric potential of which is the zeta potential. The distance between the shear plane and the nearest bulk water region is the nanoscale Debye length [60, 61]. When ionic water directionally moves along the nanochannel under an internal pressure (gravity, capillary force, etc.) or an external driver (thermal, humidity, air, etc.), the ions at the outside of the shear plane will transported together with water molecules inside the nanochannel [62, 63]. The directional ion migration along water flow results in the generation of steaming potential (Fig. 2b) [64, 65].

Different from streaming potential, ion diffusion-induce electricity is generated by establishing an ion concentration difference across the material [36, 66]. Ambient water molecules are firstly absorbed/adsorbed by materials with functional groups. The aggregation of water molecules interacts with the hydrophilic functional groups (e.g., oxygen functional groups) of nanomaterials to release mobile charged ions by breaking the polar chemical bonds to release free H^+^ [67]. The ion concentration gradient can be obtained through the asymmetric introduction of water and the nonuniform distribution of functional groups (Fig. 2c and d) [61, 68]. The dissociated ions begin to diffuse along the gradient structure, and FHG is then obtained by converting the chemical potential energy in water molecules into electricity [21].

### FHG Impact Factors

The formulas for calculating FHG are presented as follows to analyze design principles for optimizing performance. According to the following formulas, FHG performance is affected by many external and internal factors. The streaming potential-induced voltage (V_s_) and current (I_s_) are calculated with Eqs. (1) and (2), respectively [27, 69]:

1 $$V_{s} = \frac{{\varepsilon_{0} \varepsilon_{r} }}{\sigma \eta }\Delta P\zeta$$

where *ɛ*_0_ and *ɛ*_r_ are the vacuum permittivity and water permittivity, respectively. *σ* and *η* represent the conductivity and viscosity of the water, respectively. Δ*P* is the pressure difference across the nanochannel, and *ζ* is the zeta potential of the material.

2 $$I_{s} = \frac{{\varepsilon_{0} \varepsilon_{r} }}{\eta r}\Delta P\zeta A$$

where A indicates the cross-sectional area and radius of the nanochannel, respectively.

The ion diffusion-induced FHG generation can be described by Poisson and Nernst–Planck equations under appropriate boundary conditions [67, 70]:

3 $$\nabla^{2} \varphi = - \frac{F}{\varepsilon }\mathop \sum \limits_{i} z_{i} c_{i}$$

4 $$j_{i} = - D_{i} \left( {\nabla c_{i} + \frac{{z_{i} Fc_{i} }}{RT}\nabla \varphi } \right)$$

5 $$\nabla j_{i} = \frac{\partial c}{{\partial t}}$$

where φ, F, and ε denote the electric potential field, Faraday constant, and dielectric constant, respectively. *z*, *c*, *D*, and *j* refer to the valence of ionic species, ion concentration, diffusion coefficient, and ionic flux, respectively. *R* and *T* are universal gas constant and absolute temperature, respectively. The subscript of i represents different chemical species in the electrochemical system.

Optimized water movement and water properties can boost FHG performance from an external perspective. Water evaporation can be accelerated by improving solar radiation, increasing ambient temperature, and applying airflow [71, 72]. The accelerated ion transport along with water movement facilitates the flow and accumulation of counter-charges [73]. As known, water adsorption on solid materials is a physical exothermic process, the ambient energy input is conducive to overcoming the energy barrier of dissociation. However, the excessive radiation, thermal, and airflow will inhibit the immobilization of water molecules, thereby restricting electricity output [74, 75]. The FHG operation is more sensitive to variations in ambient relative humidity (RH). Under a low-humidity environment, devices are difficult to harvest ambient moisture and provide solvated ions [76]. However, the excessive RH will influence the water gradient, restrict ion migration, and eliminate wetting asymmetry [62, 77]. Hence, there is a delicate balance between device moisture content and ambient humidity for optimized FHG output.

Water properties are mainly influenced by ion sizes, ionic strengths, and pH values. According to the EDL diffusion theory, the size of hydrated ions determines the inner thickness of the EDL [78, 79]. For negatively charged materials, positive charges attracted electrons of the solid substrate to counteract net charge, increasing local hole concentration, electric potential, and higher output voltage [80]. Cations improve the aggregation degree of counter-ions and favor the enhanced surface charge density for the build-in of pseudo-streaming current. The anions with bigger sizes result in larger voltage while the sizes of cations show negligible affect [65, 81]. The Debye length is inversely proportional to the ionic strength of solutions. The increased ion concentration (brine/saline) leads to decreased Debye lengths, smaller solution dielectric constants, and enhanced surface charge densities, which eventually improves the electricity output [82, 83]. However, nanochannels will cause a shielding effect and lose charge selectivity at excessive concentrations, which leads to performance decay [84]. The pH of an aqueous solution essentially affects the dissociation of oxygen-related groups of solid substrates [85, 86]. On the one hand, acidic solutions can provide more hydrogen ions. A higher surface charge density and lower internal resistance are beneficial for voltage output. On the other hand, the ion dissociation is inhibited by the high H^+^ concentration in an acidic condition, while the dissociation is remarkably promoted in the alkaline condition [87, 88]. Performance optimization can be achieved by rational regulation of pH value.

In addition to external environmental factors, the performance can be enhanced by optimizing materials, technologies, and parameters during internal FHG system construction. The fabrication strategies and characteristics of FHG will be discussed thoroughly in the next chapter.

## Fabrication and Functions of FHG

**Fig. 3:**
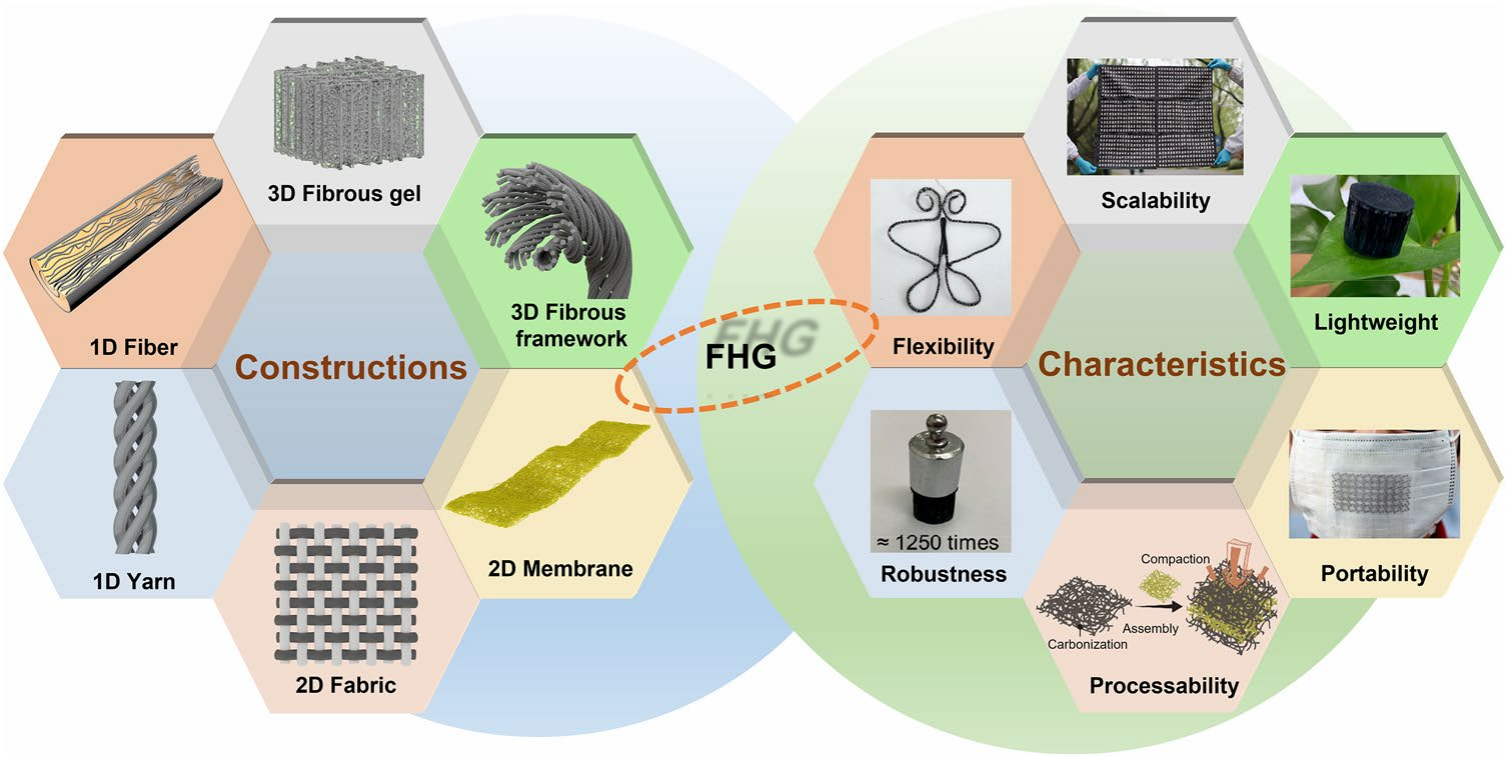
The constructions and characteristics of FHG devices. Reproduced with permission from Ref. [89], Copyright 2017, Elsevier; Ref. [90], Copyright 2018, Elsevier; Ref. [91], Copyright 2020, American Chemical Society; Ref. [92], Copyright 2024, American Chemical Society; Ref. [93], Copyright 2023, Elsevier; Ref. [94], Copyright 2024, American Association for the Advancement of Science

According to the varied compositions, constructions, and dimensions, FHG can be categorized into 1D fiber, 1D yarn, 2D fabric, 2D membrane, 3D fibrous framework, and 3D fibrous gel-based devices. Fibrous materials with outstanding processability, flexibility, robustness, lightweight, portability, and scalability guarantee practical and efficient FHG performance (Fig. 3). Fibrous materials can play different and essential roles in water harvesting, proton dissociation, ion separation, and charge accumulation during the FHG process.

### Fabrication Strategies and Characteristics of FHG

Owing to its unique processability, FHG can be fabricated with 1D fiber, 1D yarn, 2D fabric, 2D membrane, 3D fibrous framework, and 3D fibrous gel (Table 1) [95–97]. FHG devices with different dimensions and construction exhibit outstanding flexibility, robustness, lightweight, portability, and scalability [98–100].

**Table 1:** Fabrication, characteristics, and advantages comparison of common FHG systems with different constructions

Construction	Fabrication	Characteristic	Performance
1D fiber (< 1 μm)	Dry spinning	Drawing of fibers in compressed air at high temperatures (> 250 °C) and speeds (> 550 m s^−1^)	Short process, high productivity, large stretching
	Wet spinning	Generation of fiber by precipitation of polymer spinning liquid in a coagulation bath	Extensive structure, Suitable for high-performance fiber fabrication
1D yarn (1 μm–1 mm)	Single-yarn modification	Functionalized modifications on the surface, inside, or in the intermediate layers of the yarn	Efficient interaction between water and nanomaterial
	Inter-yarn entanglement	Integrating several yarns with different components or functions into single strands	Strong robustness, convenient manufacturing, rich functionality
2D fabric (centimeter size, plane structure)	Textile functionalization	Direct surface loading, chemical modification, functional group manipulation	Outstanding mechanical strength and scalability
	Yarn processing	Woven, braided, and knitted with multiple functionalized yarns	Diverse functions, specialized morphology, customized performance
2D membrane (centimeter size, plane structure)	Electrospinning	Spinning of polymer solutions or melts by forming jet streams under a strong electric field	Polymer member nanoscale filaments and pores
	Spin-coating	Coating the nanofiber film evenly on the substrate	Precise control of substrate structure and film properties
3D fibrous framework (centimeter size, stereo structure)	Stereoscopic processing	Stereoscopic fabrication with functionalized yarns or fibers	Good mechanical properties, large specific surface area, flexible structure
	Plant treatment	Chemical, physical, and biological treatment of plants	Environmentally friendly, low cost, abundant raw materials
3D fibrous gel (centimeter size, stereo structure)	Hydrogel fabrication	Formatting by chemical or physical cross-linking of hydrophilic macromolecules	Hydrophilic 3D network structure with unique water affinity
	Aerogel fabrication	Formatting by supercritical drying, pyrolysis, and ice-templating	Porous nanonetwork with good conformability and abundant ion channels

**Fig. 4:**
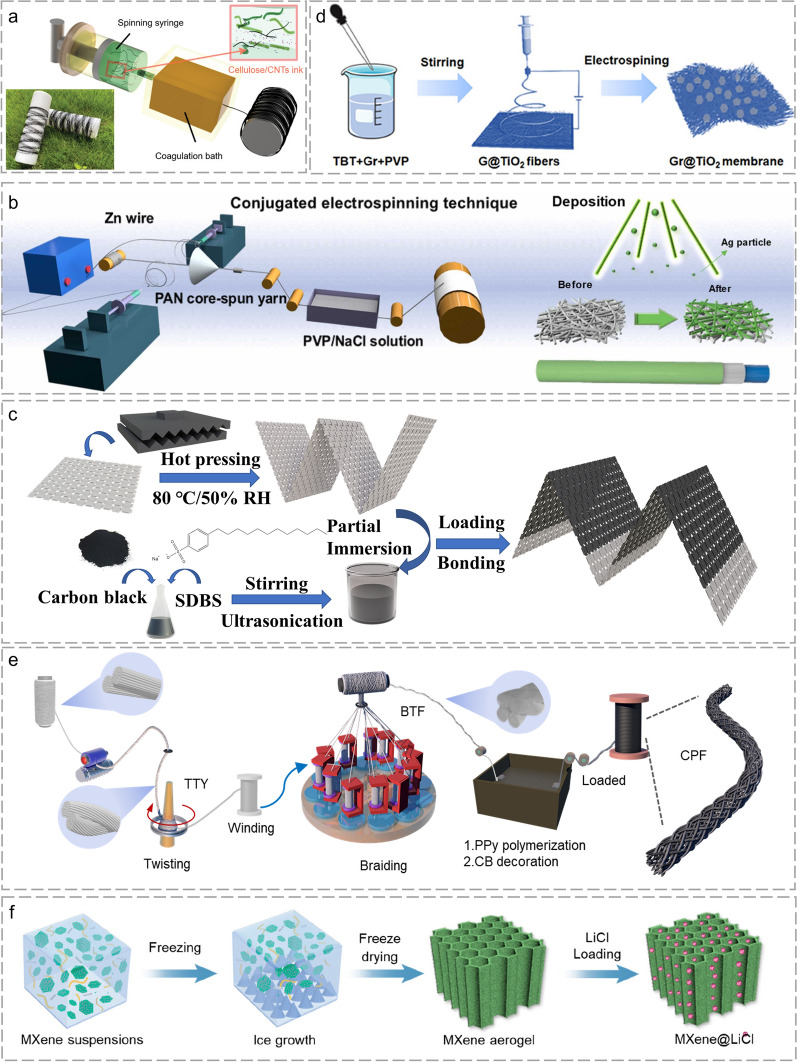
Manufacturing of fibrous materials with different constructions. The manufacturing process of **a** 1D fiber. Reproduced with permission from Ref. [101]. Copyright 2022, Wiley–VCH. **b** 1D yarn. Reproduced with permission from Ref. [102]. Copyright 2023, Elsevier. **c** 2D fabric. Reproduced with permission from Ref. [49]. Copyright 2023, Wiley–VCH. **d** 2D membrane. Reproduced with permission from Ref. [103]. Copyright 2023, American Chemical Society. **e** 3D fibrous framework. Reproduced with permission from Ref. [104]. Copyright 2024, Wiley–VCH. **f** 3D fibrous gel. Reproduced with permission from Ref. [92]. Copyright 2024, American Chemical Society

The FHG performance is closely related to the selection of raw materials and the construction of devices [105, 106]. Wet spinning is widely used to fabricate 1D fiber because of its controllable form and high production efficiency (Fig. 4a). The cellulose/carbon nanotube (CNT) fiber was reported by Chen et al. [101] When sweat was in contact with the fiber, the cellulose chain channel and spontaneous evaporation would lead to a spontaneous and continuous water flow. CNT with abundant out-of-plane π-bonds were chemically reactive with water molecules for charge separation through substantial coupling. The dissolution-solidification-regeneration system is green and environmentally friendly. It is possible to achieve the mixing and compounding of different materials to produce fibers with a variety of properties [107, 108]. 1D yarns can withstand strong mechanical bending, rolling, and twisting due to their excellent mechanical strength and flexibility [80, 109]. Yarns can be prepared by winding, braiding, and laminating technologies for multifunctional integration and mechanical enhancement [110, 111]. As shown in Fig. 4b, the hierarchical FHG with a metal wire as the core electrode, polymer-salt solution-treated nanofiber as the active layer, and decorated sliver as the shell electrode enabled rapid moisture harvesting and hydroelectricity generation. A novel core–shell structural yarn-based FHG was deformed to various shapes and easily integrated with fabrics [102]. 1D fibers and yarns can be directly manufactured into textiles with integrated HG ability and strong breathability, comfort, and fashion [112].

The HG performance of 2D fabric/membrane is generally stronger than that of 1D fiber/yarn due to the larger contact area with water. Fabrics with unique flexibility can be fabricated with a dual-active layer or single-active layer structure. The dual-active layer fabric demonstrates different polarities on both sides for gradient anionic and cationic aggregation [113, 114]. The single-active layer fabric is generally treated to provide excellent electrical conductivity, water affinity, and dense nanochannels for ion streaming [90]. In Ge et al. [49] ’s work, carbon black (CB) decorated-fabric simultaneously played the role of solar absorber for energy harvesting and a functional substance for electricity generation. Different from traditional multiple-unit integration, the all-in-one FHG device could enlarge electricity generation by increasing the wave number on a single piece of fabric (Fig. 4c). The 2D membrane is commonly fabricated through electrospinning technology with convenient procedure, wide raw materials, and rich functions [35, 115]. 2D electrospinning membrane can be prepared with co-spinning and post-loading methods. The co-spinning method mixes the active nanomaterial with the spinning solvent to prepare a pure nanofiber membrane. The nanoscale hydraulic interaction endows strong electricity generation [116]. However, the pure membrane material tends to have poor mechanical properties. Correspondingly, post-loading multilayer membranes get better robustness by sacrificing all-in-one pore distribution [102, 117]. As shown in Fig. 4d, the hydrovoltaic effect of semiconductor materials was investigated with an electrospinning membrane. The cations preferentially adsorb and form an EDL with the electrons inside the fibrous membrane [103]. 2D electrospinning membrane with abundant functional groups, interconnected porous structures, and numerous nanoscale channels enables the coexistence of streaming potential and ion diffusion [93].

The 1D and 2D fibrous substrate can also be fabricated into a 3D fibrous framework with richer internal ion channels for power enhancement. On top of that, the internal hydraulic interaction is subject to less environmental interference (humidity, airflow, temperature, etc.) [118, 119]. In Ge et al. [104]’s work, a 3D fibrous framework with tunable water flow was fabricated through twisting and braiding technology. The modified water flow rate/height/ and content ensured rapid ion migration, appropriate wetting boundary, and sufficient brine circulation, respectively. The FHG output was enhanced through structure-engineering, evaporation-acceleration, and desalination-integration (Fig. 4e). Natural wood with rich fibrils and lignin is another typical 3D fibrous framework. The removal of internal lignin and hemicellulose by chemical treatment allows the release of microns and nanopores in the cell wall, resulting in a highly porous structure [87, 120]. The chemical modification leads to the attachment of rich polar functional groups (carboxyl groups, amine groups, etc.) inside the channels for hydraulic interaction [121, 122]. The 3D fibrous gel fabricated by Cai et al. [92] through ice templating exhibited a wide specific area, high porosity, and robust skeleton (Fig. 4f). The outstanding performance could be attributed to its dual H-bond network, cellulose nanocrystal, and an ingeniously aligned wood-like channels. A considerable FHG could be achieved during water harvesting. Cellulose nanofibers are commonly selected together with polymers as raw materials for the preparation of gels through directional freeze casting, breath figure method, and cross-linking polymerization [123, 124]. The aligned nano-channels and hygroscopic units facilitate the rapid transfer of water, protons, and ions [125].

Flexibility endows FHG devices with deformable shapes, adjustable sizes, and varied performances. Different shapes can be prepared by manual bending and machine processing [34]. Different morphologies (length, width, thickness, etc.) of FHG devices can be manufactured with varied properties by conveniently cutting, sewing, and splicing [139, 140]. The robustness of FHG devices is evaluated by tensile strength, tensile modulus, flexural strength, fatigue strength, and so on [141, 142]. The strong robustness relies on the optimized material selection, processing procedures, and finishing methods, which is vital for durable operation in harsh and cyclic conditions [143, 144]. The density of most fiber/yarn is less than 2 g cm^−3^ [112, 145]. Compared with common metal (oxide)-based HG devices, FHG with superior lightweight characteristics facilitates the integration with masks, clothes, umbrellas, and tents. These portable and practical devices promote the development of smart textile equipment [45]. Due to the long history of the textile industry and mature manufacturing streamlines, FHG devices can be fabricated and integrated at scale with commercial technologies including weaving, knitting, braiding, electrospinning, melt blown, and so on [146–148]. Fibrous materials with rich fabrication strategies and varied constructions provide a solid foundation for efficient FHG performance (Table 2).

**Table 2:** Performance comparison of recently reported FHG systems

Construction	Material	Current	Voltage (V)	Power	References
1D fiber	GO fiber	1.06 mA cm^−2^	0.355	–	[89]
	Silk nanofibrils	–	0.120	–	[126]
	Silk cocoon	62 μA	0.436	–	[127]
	CNT/cellulose fiber	171.8 nA	0.160	0.4 mW cm^−3^	[101]
1D yarn	GO coaxial yarn	10 μA	0.3	0.21 μW cm	[90]
	MoS_2_/carbon yarn	2 mA	0.54	10.8 W cm^−2^	[128]
	CNT yarn	83 μA	0.31	30 mW cm^−2^	[65]
	CNT/CB yarn	29.5 μA	0.43	5.833 mWh cm^−3^	[80]
2D fabric	MoS_2_/fabric	0.27 mA cm^−2^	0.8	36.12 μW cm^−2^	[129]
	CB/cellulose	100 μA	0.3	4.58 μW	[130]
	MXene/CNT/cotton	520 μA	0.36	46.63 μW	[86]
	Metal/bacteria cellulose	7.51 mA	0.935	6.07 mW	[131]
2D membrane	PAN/PSSA membrane	1.35 μA cm^−2^	1.1	1.48 μW cm^−2^	[132]
	Cellulose acetate membrane	3.5 μA	0.7	2.45 μW cm^−2^	[119]
	Silk/nylon	130 nA	4.82	–	[116]
	Carbonized Polyacrylonitrile	9.36 μA	0.282	83 nW cm^−2^	[133]
3D fibrous framework	Delignified wood	6 μA	0.14	1.35 μW cm^−2^	[87]
	PEDOT: PSS/wood	11 μA	0.385	198 nW	[134]
	Carbonized wood	10.5 μA	0.096	287 nW	[135]
	PPy/CB/Tencel	0.6 μA	0.73	0.168 μW	[104]
3D fibrous gel	Nanofibrils aerogel	45 nA	0.115	–	[44]
	Cellulose aerogel	30 nA	0.11	0.63 nW cm^−2^	[136]
	Graphene aerogel	0.5 mA	0.87	6.84 μW cm^−2^	[137]
	GO-CNF aerogel	60 μA	0.55	0.82 mWh cm^−3^	[138]

### Advanced Functions of FHG

Various fibrous materials with different constructions, characteristics, and advantages have been developed for FHG. Generally, there are 4 steps including water harvesting, proton dissociation, ion separation, and charge accumulation for efficient and durable hydrovoltaic generation [22, 31, 43]: (1) Fibrous materials with abundant hydrophilic functional groups harvest water from the environment. (2) The aggregation of water molecules causes proton dissociation, which breaks the polar chemical bonds and releases numerous charged ions. (3) The immobilized and charged functional groups drive the movement of counter-charged ions as carriers. The released ions are separated and transported under the built-in electric field. (4) The counter-charged ions are accumulated on the opposite electrodes. The ionic current is converted to electronic current via an electrochemical reaction. The detailed description of the advanced functions is presented as follows:

#### Water Harvesting

The water affinity and sorption site are the key factors in water harvesting. The hydrophilicity directly affected the accessibility and motion state of water molecules inside the fibrous materials [149]. A large surface area provides abundant sorption sites, increased functional groups, and improved ion/electron interaction area. Enhanced water sorption will further increase available ionized water content for transport and electrochemical reactions [43]. Besides, fibers can provide a directional track for droplet sliding due to the 1D structure and low pinning effect, which allows rapid water transfer along the axial direction. Thus, the slender structure offers sufficient liquid–solid contact area for electrostatic induction [150, 151]. Water tends to apply on the nanofiber surface with sufficient contact area. Meanwhile, the side area helps capture water along with the fiber orientation [133].

**Fig. 5:**
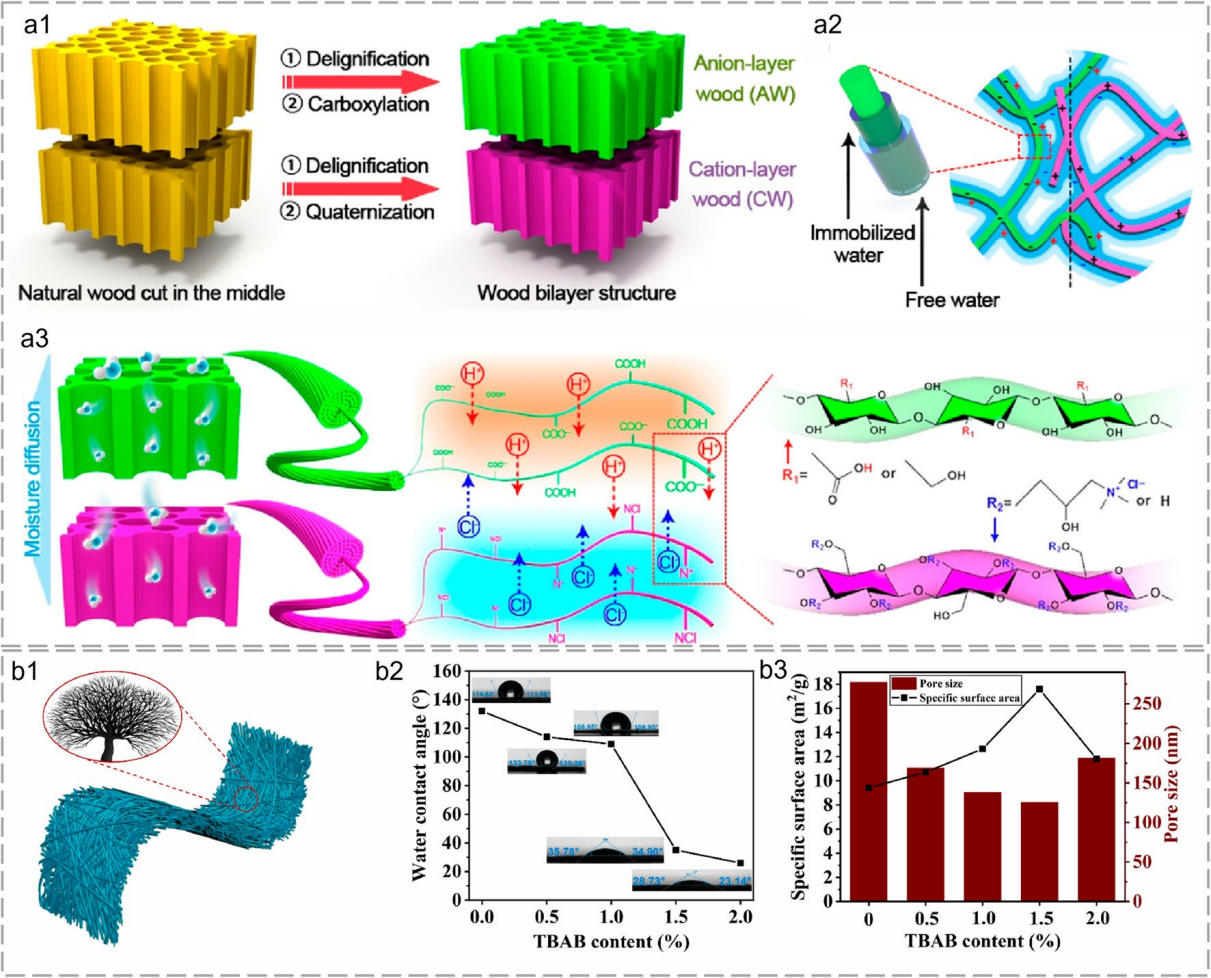
Fibrous materials for water harvesting during FHG. **a1** Schematic of the design concept and fabrication process. **a2** Schematic of FHG enabled by directional migration of free ions with opposite charges within hydrated nanochannels. **a3** Graphical illustration of FHG process. Reproduced with permission from Ref. [75]. Copyright 2022, American Chemical Society. **b1** Schematic of the tree-like fibrous membranes. **b2** Water contact angle of the fibrous membranes with different TBAB content. **b3** Specific surface area and average pore size of the fibrous membranes with different TBAB content. Reproduced with permission from Ref. [119]. Copyright 2022, Elsevier

A cellulose wood-based generator was fabricated after delignification and chemical modification by Cai et al. (Fig. 5a1). The treated wood exhibited a robust framework with natural transpiration pathways and aligned ion nanochannels for spontaneous moisture adsorption (Fig. 5a2). The hydrophilic groups of wood immobilized water molecules through hydrogen bonds to form hydrated nanochannels. Lignin was dissolved and micro/nanoscale voids were formed after delignification (Fig. 5a3). The increased specific surface area (from 2.5 to 14.8 m^2^ g^−1^) provided aligned micro/ nanoscale channels and abundant active sites for interaction with water. The increased water content assisted the transport and release of ions, thereby improving electricity generation performance. The enhanced water adsorption at high humidity of 75% generated a steady output voltage of around 0.45 V for over 24 h [75, 87]. A tree-like structured nanofiber membrane was fabricated by Zhang et al. through one-step electrospinning (Fig. 5b1). The structural properties of fibrous materials including specific surface area, surface hydrophilicity, and pore structure were vital for water harvesting [152]. The hydrophilicity and specific surface area of fibrous membranes were tuned by adding organic branched salt (tetra butyl ammonium bromide, TBAB) to the spinning solution (Fig. 5b2 and b3). The membrane hydrophilicity was enhanced in a gradient after adding TBAB. The specific surface area was optimized from 9.42 to 17.59 m^2^ g^−1^ by variation of the tree-like nanofibers content. The improved hydrophilicity and specific surface area facilitated water molecules harvesting and ion migration within the membrane. Therefore, an output voltage of about 0.7 V and a power density of 2.45 μW cm^−2^ was obtained at room temperature [119].

#### Proton Dissociation

The proton dissociation ability mainly depends on the surface property. The oxygen-containing groups are prone to adsorb water molecules and release numerous protons [74]. The fibrous materials with abundant and diverse functional groups (such as hydroxyl, carboxyl, carbonyl, and sulfonic groups) may release hydrogen ions when interacting with water [153]. Then, a bilayer-hydrated shell combined with an inner layer with immobilized water molecules and an outer layer with free water molecules is generated. Afterward, dissociable groups tend to form hydrated and charged nanochannels for directional ion migration with the presence of EDL [154]. The surface of fibrous materials is charged after being hydrated. The zeta potential dictates the ion polarity, ion potential, and Debye length. A higher magnitude of zeta potential increases electrostatic repulsion ability [93]. Materials with opposite zeta potential attract divergent charge carriers, thus generating electrical outputs with reversed polarity [36, 69]. Besides, fibrous materials with strong chemical potential and rich specific surface area favor proton dissociation through functional group hydrolysis [25].

**Fig. 6:**
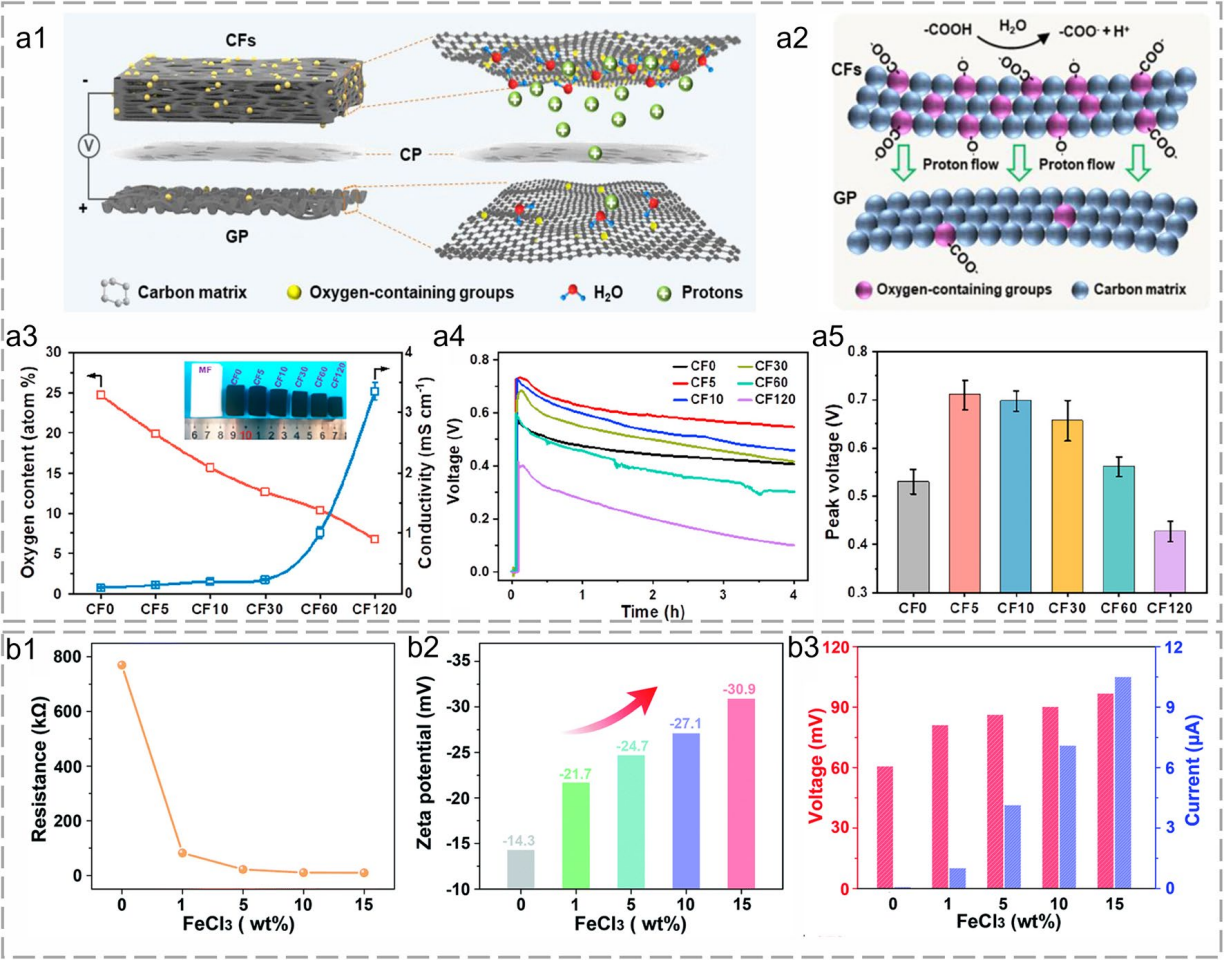
Fibrous materials for proton dissociation during FHG. **a1** Schematic diagram of a self-supporting bilayer generator. **a2** Schematic mechanism of the bilayer generator. **a3** Variations of oxygen content and conductivity of carbon foams with carbonization time at 800 °C (inset, digital photograph of carbon foams). **a4** Real-time open-circuit voltages. **a5** Peak voltages induced by carbon foams in water. Reproduced with permission from Ref. [74]. Copyright 2024, American Chemical Society. **b1** Resistance, **b2** zeta potential, and **b3** FHG output of the wood-based generator under different concentrations of FeCl_3_ solution. Reproduced with permission from Ref. [135]. Copyright 2022, Royal Society of Chemistry

In Xue et al.’s research, a self-supporting bilayer electric generator was fabricated with carbon foam and graphite paper (Fig. 6a1). The varied polar groups' amount and surface proton dissociation of carbon foam and graphite paper created a potential difference (Fig. 6a2). The surface properties were regulated reliably by adjusting the pyrolysis parameters. The enhanced conductivity was attributed to the reduction of oxygen and structure defects as well as the growth of graphitic N species. Meanwhile, the shortage of oxygen-containing groups resulted in less hydrolysis [155]. The increased oxygen-containing groups enhanced proton dissociation, while increased conductivity facilitated ion migration (Fig. 6a3). There was a balance between the oxygen content and the conductivity for optimized performance, which can be regulated by reduction and oxidation processes (Fig. 6a4 and a5) [74]. A conductive carbonized cellulose wood with abundant hydroxyls was fabricated by Zhang et al. via a Lewis acid metal salt-catalyzed carbonization process (Fig. 6b). Acid degraded the fiber in both the amorphous and qualitative regions, hydrolyzed the glucose unit into a short chain, and reduced the cellulose crystallinity [156]. A dramatic increase in carbonization led to reduced electrical resistance (from 769 to 9.411 kΩ) (Fig. 6b1). The decreased resistance (increased conductivity) improved the current output. Meanwhile, the increased zeta potential owing to elevated acid concentration meant a higher negative charge, which improved the voltage output (Fig. 6b2) [135]. Hence, a stable voltage output of 96 mV and a current output of 10.5 mA was stability generated over 24 h under ambient conditions (Fig. 6b3).

#### Ion Separation

The ions dissociated from water molecules move along the chemical or mechanical gradient for FHG. As for the ion diffusion-induce generator, the charge carriers are electrostatically coupled to the mobile ions, leading to charge transfer along the chemical gradient. The chemical gradient can be obtained from the functional group gradient, ionic gradient, and humidity gradient [153, 157]. The Janus or multi-layer construction generator with varied functional groups exhibits large or opposite surface charges, which can enhance ion-directed migration and suppress charge recombination [66]. The asymmetric charged generator can be fabricated through gradient functionalization or ion doping. The dopant ions will migrate with the diffused water and boost electrical output [158]. Moist may serve as a trigger for establishing charge concentration gradient under varied RHs. Mechanical gradient mainly occurs for streaming potential-induced FHG. The charged fibrous materials with porous or micro-channel structures exhibit strong capillary pressure [159]. Hydrodynamic flow driven by a pressure gradient between the narrow channels causes directional counterions migration, thereby generating a stable potential difference between two ends of the nanochannel [56]. The varied internal porosity, pore size, and tortuosity cause a huge effect on capillary pressure, which is vital for ion transfer and streaming potential.

**Fig. 7:**
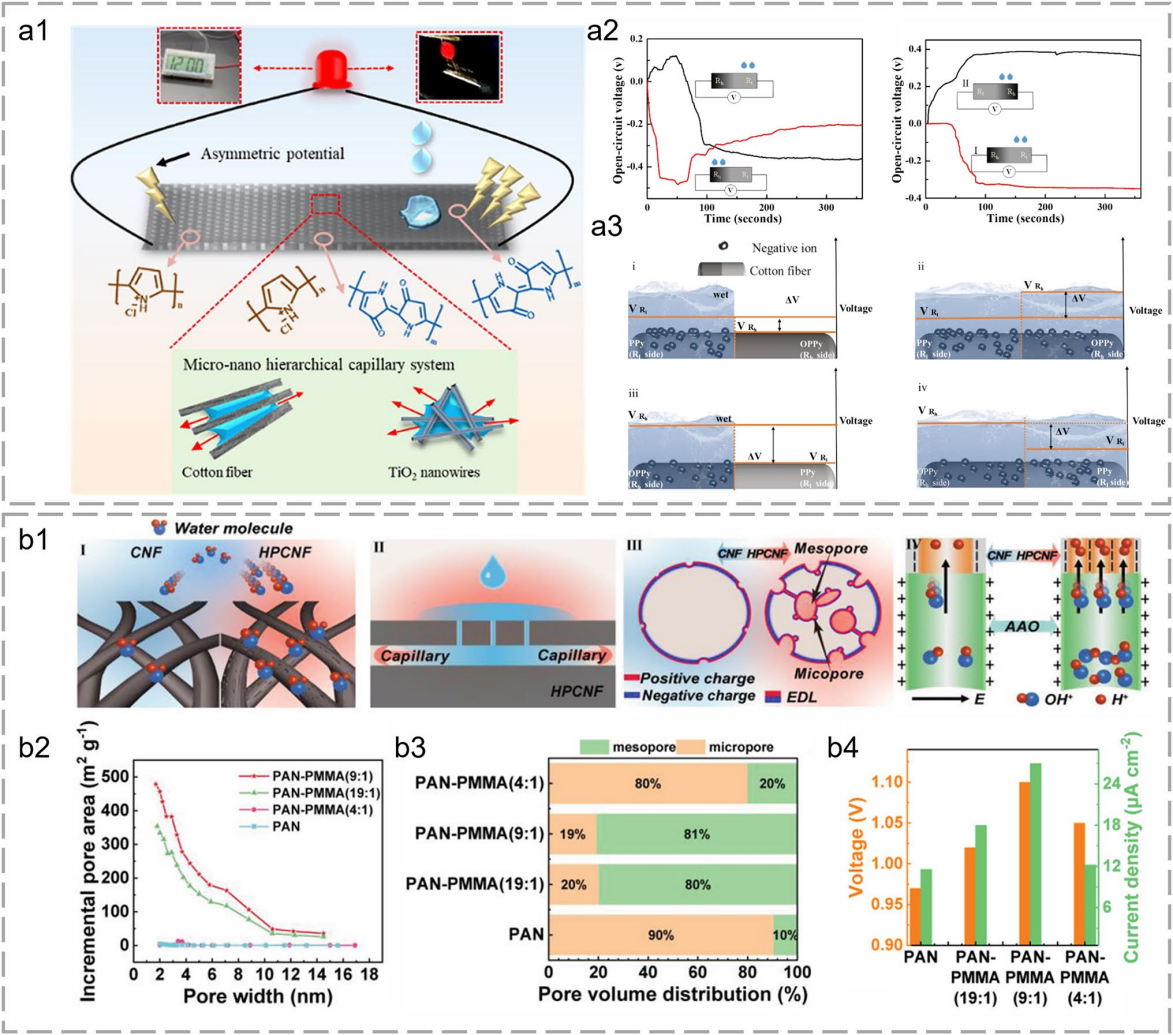
Fibrous materials for ion separation during FHG. **a1** Schematic diagram of an asymmetric hierarchical textile for FHG. **a2** Voltage in response to dropping water onto the asymmetric textile with the resistance of 200 kΩ (left) and 50 kΩ (right). **a3** Schematic illustration of the establishment of a potential difference between the asymmetric areas when half-wetted and fully-wetted. Reproduced with permission from Ref. [157]. Copyright 2022, Elsevier. **b1** Schematic diagram of four functions of hierarchical porous nanofibers. **b2** Incremental hole area versus hole width curve. **b3** Proportion of mesopores and micropores in hierarchical porous nanofibers with different PAN-PMMA ratios. **b4** FHG output of devices with different PAN-PMMA ratios. Reproduced with permission from Ref. [43]. Copyright 2022, Wiley–VCH GmbH

A textile-based asymmetric hierarchical system was fabricated by Xie et al. for constant FHG through the corporation of ion diffusion and streaming potential (Fig. 7a1). The textile exhibited a micro-nano hierarchical capillary system and asymmetric polypyrrole (PPy) loading. The micro-nano channel formed on adjacent cotton fibers and yarns promoted water transport and ion separation, increasing the output value through streaming potential. The ion diffusion-induced FHG was highly influenced by varied contents of functionalized loading with different resistances (Fig. 7a2). A potential was raised immediately when a droplet contacts with asymmetric textiles. Hydronium ions flowed downstream and generated a positive electrokinetic potential under capillary action in the charged nanochannels. Stable power output was sustained through ion diffusion when fully wetted owing to the chemical gradient across the textile (Fig. 7a3) [157]. In Lu et al.’s research, hierarchical porous carbon nanofibers were fabricated through electrospinning technology (Fig. 7b1). The pore distribution and density were modified through spinning solution characteristics, spinning parameters, and chemical post-modifications (Fig. 7b2). The hierarchical porous structure combined with macropores (> 50 nm), mesopores (2–50 nm), and micropores (< 2 nm) reduced the transport resistance and accelerated the water diffusion (Fig. 7b3). Hierarchical pores also extended the three-phase contact line between water and fibrous substrate, enhanced the capillary force, and flattened the curved droplet surface. Faster water transport enabled promoted ion migration, ensuring higher power output. Hierarchical pores formed a strong built-in electric field for efficient selective separation and transport of ions. Besides, the channel thickness also influenced molecular junctions and intrinsic resistances. In general, there was a delicate balance between pore sizes and specific surface areas for enhanced FHG performance (Fig. 7b4) [43, 117, 123].

#### Charge Accumulation

**Fig. 8:**
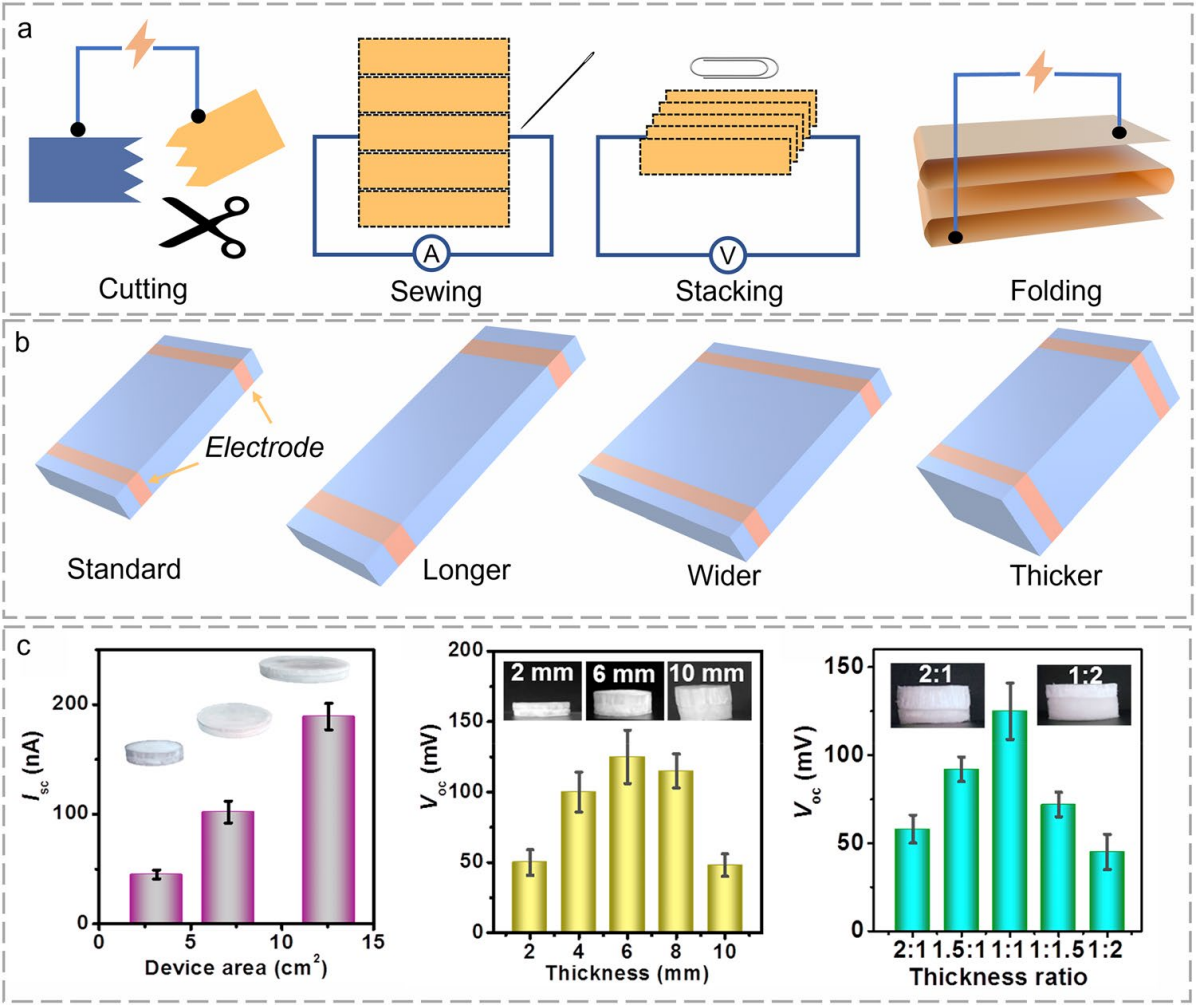
Fibrous materials for charge accumulation during FHG. **a** Schematic diagram of the manufacturing of flexible FHG device. **b** Schematic diagram of the FHG device with varied geometric morphology of flexible FHG device. **c** Effect of aerogel geometric morphology on electricity output. Reproduced with permission from Ref. [44]. Copyright 2021, Elsevier

During the charge accumulation process, geometric structures play a prominent role in electricity output. FHG devices can be cut, sewn, stacked, and folded into various morphologies owing to their outstanding flexibility (Fig. 8a) [84]. The varied lengths, widths, and thicknesses lead to different charge accumulation and electricity generation performances (Fig. 8b) [135, 160]. A higher length means a larger surface area for charge absorbing and accumulation, resulting in a larger potential gradient. The current first improves with the length because of increased free ions content. However, the tedious length causes restricted capillary wicking forces, excessive resistances, and elongated transmission paths [132, 133]. As the width improves, current increases continuously due to growing water transport channels and evaporation surface areas. However, overhigh widths make it difficult to achieve a balance between evaporated water and replenished water, thereby weakening the current [75, 134]. The increased thickness leads to more intense nanochannels and enhanced water content for stronger output. However, excessive thickness causes weak moisture infiltration and disordered ion movement because the evaporation process mainly exists on the interface layer. Besides, excessive ion content results in ionic erosion damage and durability decay [126, 161, 162]. In Yang et al.’s research, geometric structures of the fibrous aerogels were modified for optimal charge accumulation (Fig. 8c). Convenient and flexible manufacturing processes guarantee optimized structural engineering. Hence, comprehensive consideration of the FHG performance, manufacturing difficulty, and cost-effectiveness is vital for the geometric selection [44, 113].

Overall, fibrous materials with different constructions possess advanced functions including water harvesting, proton dissociation, ion separation, and charge accumulation. The FHG performance can be optimized through material refinement, surface treatment, interface engineering, and morphology modification [14]. The rational regulation of external factors and internal characteristics are both essential for FHG efficiency enhancement.

## Applications of FHG

Many applications of FHG have been presented due to water ubiquity, flexible construction, and promising performance. FHG devices can be applied for energy utilization and regeneration like other HG equipment [163, 164]. Moderate and continuous seawater flow during FHG can also be simultaneously utilized for photothermal desalination. For instance, a CNT fabric electrode was fabricated by Song et al. for sustainable water self-pumping, excellent conductivity, and intense radiation harvesting. The synergistic utilization of solar desalination and FHG would also unchain the bottleneck of weak and discontinuous electrical outputs [48, 165]. Recently, several interesting applications including power supply, energy storage, electrical sensor, and information expression have been realized through FHG with unique flexibility and wearability [166, 167]. The development of these advanced applications paves the way for the potential utilization of the Internet of Things (IoT) [168–170].

**Fig. 9:**
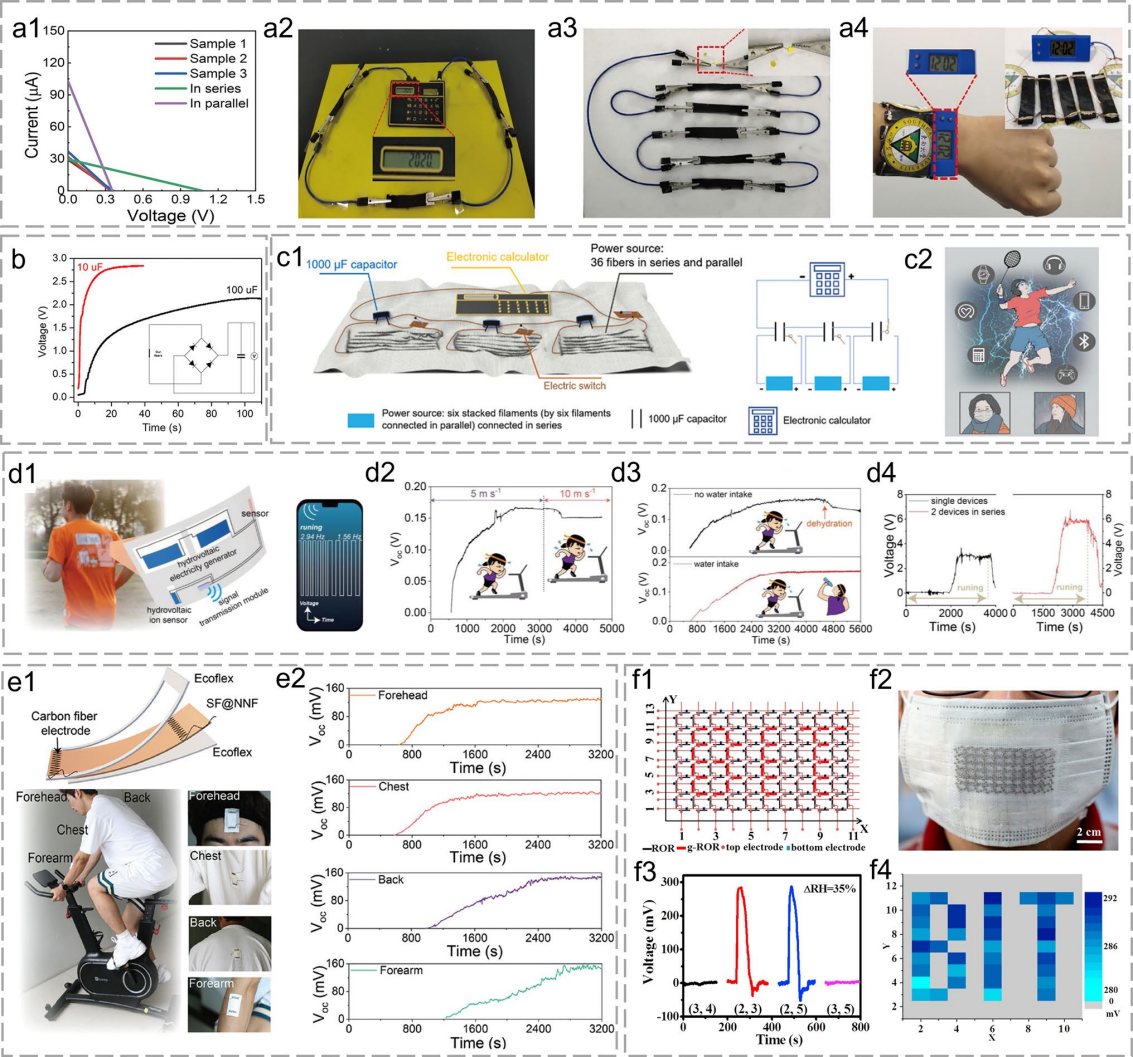
Applications of FHG. **a1** Measured electricity output of three paper-based generators connected in parallel or series. Photograph of **a2** an electronic calculator, **a3** an LED bulb, and **a4** a wearable watch driven by arrayed connected generators. Reproduced with permission from Ref. [88]. Copyright 2020, Elsevier. **b** Voltage–time curves of commercial capacitors charged by fluidic nanogenerators connected in series. Reproduced with permission from Ref. [65]. Copyright 2021, American Chemical Society. **c1** The device containing CNT fibers is woven into the fabric. **c2** A series of potential applications for FHG devices. Reproduced with permission from Ref. [101]. Copyright 2022, Wiley–VCH GmbH. **d1** Schematic diagram of a wearable self-powered sensing system. Real-time V_oc_ variation of the FHG ion sensor during **d2** running at different speeds and **d3** running with and without water intake. **d4** Real-time V_oc_ curves of wearable single and two FHG devices in series generated during running. Reproduced with permission from Ref. [174]. Copyright 2023, Wiley–VCH GmbH. **e1** Schematic diagram of the FHG structure and attachment position for wearable sweat sensing. **e2** Real-time V_oc_ variation of different positions during constant riding. Reproduced with permission from Ref. [116]. Copyright 2024, Wiley–VCH GmbH. **f1** Schematic illustration of unit coordinates. **f2** The device containing 136 units is attached to a mask. **f3** Peak voltage outputs of the units located in different coordinates. Expression of the electronic label: voltage outputs of all the units after a deep breath (ΔRH = 35%). Reproduced with permission from Ref. [89]. Copyright 2017, Elsevier

FHG with different constructions demonstrates outstanding electricity output performance. However, it is difficult for a single FHG device to directly drive the operation of electronic components [171]. As shown in Fig. 9a1, several FHG units were usually connected in series/parallel into an integrated powering array for practical power supply. A flexible and cost-effective ($ 0.089) paper-based power generator was fabricated by Lyu et al. The electricity output could be easily enlarged through series and parallel connections. The voltage enhancement was easily achieved through multi-layer stacking in series. An 8-digit electronic calculator was powered with three devices connected in series (~ 1.076 V and ~ 29.6 μA) (Fig. 9a2). Yellow light-emitting diodes (LED) with a minimum working voltage of 1.8 V and a current of 100 μA were lighted by the integrated device (Fig. 9a3). Besides, the flexible device could also act as an excellent candidate for powering wearable watches (Fig. 9a4) [88].

The discontinuity and sensitivity during FHG may affect stable electricity outputs. The electricity generated by FHG devices can be conveniently accumulated and stored with commercial capacitors [172]. In Zhao et al.'s research, a commercial capacitor could be charged up to 2.6 V (10 μF) within 10 s or 2 V (100 μF) within 100 s by fiber-based devices (Fig. 9b) [65]. Acidified CNT fibers in series or parallel were woven into fabrics to charge 1000 µF capacitors by Chen et al. [101] (Fig. 9c1). A 0.4 V voltage was charged within 10 min for energy storage. Capacitors were connected in series to power IoTs such as calculators, earphones, medical sensors, and cell phones with renewable water sources including sweat, tears, and rainwater (Fig. 9c2).

Robust FHG devices can maintain the electric output under vigorous bending and friction conditions. Hence, deformable FHG devices can be easily installed into clothes or wearable garments as electrical sensors without destroying the appearance [173]. Zhang’s group utilized sweat to simultaneously build a wearable power supply for multiple sensors and realized sweat ion sensing for sports and health through FHG (Fig. 9d1). The Cl^−^ concentration in sweat was closely related to the sweating rate. The voltage signal variation was approximately corresponding to Cl^−^ concentration because Cl^−^ was the most abundant inorganic anion to trigger FHG. Dehydration may occur after prolonged running or high-speed running (Fig. 9d2). The monitoring of Cl^−^ through Bluetooth wireless signal transmission module provided an important basis for balancing body fluid ions (Fig. 9d3). The array amplification provided diverse practical utilization scenarios (Fig. 9d4) [174, 175]. Zhang’s group also attached the wearable FHG sensor to the chest, back, forehead, and forearm to monitor sweat and the extent to which different body parts sweat (Fig. 9e1). The motion state was related to the electrolyte ion concentration in the sweat, FHG sensors were used as a potential way for sweat health monitoring. Different voltage response speeds and strengths demonstrated the varied perspiration rates and amounts of different body parts during exercise (Fig. 9e2) [116, 176, 177].

Functional fibers/yarns can be weaved, knitted, and processed into textiles to simultaneously realize wear comfort and information expression [110, 178, 179]. In Liang et al. [89]’s research (Fig. 9f1), functional graphene oxide fibers with varied moisture sensitivities were considered as the “0” (OFF, low voltage) and “1” (ON, high voltage) status because of different voltage outputs. The elements “0” and “1” formed the fundamental informational units of an electronic label. The mask's unit count was changed based on the complexity of the information expression (Fig. 9f2). The mask could express 2^136^ kinds of different information with 136 information units under breath (Fig. 9f3). Once the human took a deep breath (ΔRH = 35%), the varied voltages of the units located in different coordinates led to diverse information expression (Fig. 9f4).

Overall, FHG demonstrates extensive and unique functions including water treatment, power supply, energy storage, electrical sensor, and information expression. In particular, the integration with wearable electronics will be a key research and development direction for FHG with special lightness, flexibility, and customizability [180–182]. Therefore, it is crucial to further enhance wearing comfort, mechanical robustness, washing resistance, dyeability, stylishness, portability, and electrical safety while maintaining hydrovoltaic performance. Fibrous materials need to be upgraded in terms of material modification, process optimization, and functional innovation to cope with novel applications.

## Conclusion and Perspective

Water is Earth’s largest potential energy source. The exploration and development of water energy can greatly alleviate the energy crisis, which is of great ecological value and social significance. HG with unique environmental-friendliness and cost-effectiveness has been widely applied for converting energy from ubiquitous water. The development of HG based on extensive materials has proved to be of great environmental and energy significance. Fibrous materials with unique flexibility, processability, multifunctionality, and practicability play varied roles in efficient FHG. In particular, FHG devices hold unique prospects for constructing wearable electronic equipment owing to their comfort, lightness, breathability, and softness. Fibrous materials with different constructions exhibit advanced functions during water harvesting, proton dissociation, ion separation, and charge accumulation processes. Optimizations at every step of the FHG process are critical to improve performance and practicality. There is a strong requirement for deep investigation including atomic modification, molecular processing, interfacial engineering, and structural tailoring. In addition, it is also crucial to pay attention to the environmental conditions according to the actual application scenario.

Despite the outstanding advantages and considerable achievements, lots of challenges remain to be addressed.

1. Further in-depth and detailed mechanistic investigations. Although streaming potential and ion diffusion mechanisms have been proposed for FHG analysis, there are still many confusing phenomena and beyond-limit outputs. The atomistic interaction between functional materials and water molecules is rarely explored. Hence, advanced characterizations and sophisticated experiments should be conducted to expose deep and sound operational mechanisms.
2. General and universal measurement standards. As mentioned above, FHG performance is affected by abundant external factors including humidity, temperature, and water properties. Internal material composition, construction, and morphology also show huge effects on power generation. Active electrode reactions may cause massive interference in performance comparisons. Hence, general standards to evaluate output performance are extremely important. The standardization of measurement procedure, quantitative energy input, and precise FHG output should be meticulously performed.
3. Diverse system construction and in-depth characteristic exploration. The integration of fibrous materials with carbon materials is widely used due to convenient preparation processes. However, the carbon–water interaction is inherently weak and limited to the surfaces of atomic layers. Hence, rational material selection and in-depth characteristic exploration need to be adopted. Fibrous materials are commonly selected as aqueous substrates, but the microscopic interaction is often overlooked. The nanoscale fibrous structures are desired to expand interaction level, scope, and depth.
4. Enhanced, efficient, and sustainable electricity output. The millivolt-level voltage output and microampere-level current output are still far from the daily application. The selection and processing of fibrous materials can be adopted through materials optimization and structure engineering for enhanced energy conversion. Arrayed integration should be conducted with optimal simplicity and convenience. It requires greater homogeneity of fibrous materials, improved connections between devices, and optimized device layout. Continuous and moderate water supply should also be optimized for spontaneous and prolonged operation. In addition, improving the lifespan and robustness of fibrous materials is also beneficial for sustainable hydrovoltaic generation.
5. Expanded application scenarios. Energy utilization and regeneration through water sources are important scopes for the development of HG. Fibrous materials with unique flexibility and wearability should concentrate more on the construction of wearable electronics. The body's metabolism including breath, sweat, and heat from the human body is an ideal trigger for sustainable FHG operation. The research and development of wearable textiles are crucial for broader FHG applications. The interdisciplinary collaborations including artificial intelligence, machine learning, sports health, and informatics transmission will offer distinctive applications.
